# Natural *Leishmania* (*Viannia*) spp. infections in phlebotomine sand flies (Diptera: Psychodidae) from the Brazilian Amazon region reveal new putative transmission cycles of American cutaneous leishmaniasis

**DOI:** 10.1051/parasite/2016022

**Published:** 2016-05-27

**Authors:** Adelson Alcimar Almeida de Souza, Thiago Vasconcelos dos Santos, Yara Lúcia Lins Jennings, Edna Aoba Yassui Ishikawa, Iorlando da Rocha Barata, Maria das Graças Soares Silva, José Aprígio Nunes Lima, Jeffrey Shaw, Ralph Lainson, Fernando Tobias Silveira

**Affiliations:** 1 Parasitology Department, Evandro Chagas Institute (Surveillance Secretary of Health, Ministry of Health) Ananindeua Pará State Brazil; 2 Tropical Medicine Nucleus, Federal University of Pará Belém Pará State Brazil; 3 Biomedical Sciences Institute, São Paulo University São Paulo State Brazil

**Keywords:** *Leishmania* (*Viannia*) spp., Phlebotomine sand flies, Natural infections, Amazon region, Pará State, Brazil

## Abstract

In Amazonian Brazil the etiological agents of American cutaneous leishmaniasis (ACL) belong to at least seven *Leishmania* species but little is known about the putative phlebotomine sand fly vectors in different biomes. In 2002–2003 a survey of the phlebotomine fauna was undertaken in the “Floresta Nacional do Tapajós”, Belterra municipality, in the lower Amazon region, western Pará State, Brazil, where we recently confirmed the presence of a putative hybrid parasite, *L.* (*V*.) *guyanensis × L.* (*V.*) *shawi shawi*. Sand flies were collected from Centers for Disease Control (CDC) light traps, Shannon traps and by aspiration on tree bases. Females were dissected and attempts to isolate any flagellate infections were made by inoculating homogenized midguts into Difco B^45^ medium. Isolates were characterized by monoclonal antibodies and isoenzyme electrophoresis. A total of 9,704 sand flies, belonging to 68 species or subspecies, were collected. Infections were found in the following sand flies: *L.* (*V.*) *naiffi* with *Psychodopygus hirsutus hirsutus* (1) and *Ps. davisi* (2); and *L.* (*V.*) *shawi shawi* with *Nyssomyia whitmani* (3) and *Lutzomyia gomezi* (1). These results provide strong evidence of new putative transmission cycles for *L.* (*V.*) *naiffi* and *L.* (*V.*) *s. shawi.*

## Abstract (fr)

En Amazonie brésilienne les agents étiologiques de la leishmaniose cutanée américaine (LCA) appartiennent à au moins sept espèces de *Leishmania*, mais on connaît mal les phlébotomes vecteurs dans les différents biomes. En 2002 et 2003 une enquête sur la faune des phlébotomes a été entreprise dans la « Floresta Nacional do Tapajós », commune de Belterra, dans la région inférieure de l’Amazonie, ouest de l’État du Pará, au Brésil, où nous avons récemment confirmé la présence d’un parasite hybride putatif, *L.* (*V.*) *guyanensis* × *L.* (*V.*) *shawi shawi*. Les phlébotomes ont été recueillis dans des pièges lumineux CDC, des pièges Shannon et par aspiration sur la base des arbres. Les femelles ont été disséquées et on a tenté d’isoler les infections par des flagellés en inoculant des mésogastres homogénéisés dans un milieu Difco B45. Les isolats ont été caractérisés par des anticorps monoclonaux et l’électrophorèse des isoenzymes. Un total de 9704 phlébotomes, appartenant à 68 espèces ou sous-espèces, ont été collectés. Des infections ont été trouvées dans les phlébotomes suivants : *L.* (*V.*) *naiffi* avec *Psychodopygus hirsutus hirsutus* (1) et *Ps. davisi* (2); *L.* (*V.*) *shawi shawi* avec *Nyssomyia whitmani* (3) et *Lutzomyia gomezi* (1). Ces résultats fournissent des preuves solides de nouveaux cycles putatifs de transmission pour *L.* (*V.*) *naiffi* et *L.* (*V.*) *s. shawi*.

## Introduction

In the Brazilian Amazon region, American cutaneous leishmaniasis (ACL) is caused by at least seven *Leishmania* species that are dermotropic in man, namely: *Leishmania* (*Viannia*) *braziliensis* Vianna 1911, *L.* (*V.*) *guyanensis* Floch 1954, *L.* (*Leishmania*) *amazonensis* Lainson and Shaw 1972, *L.* (*V.*) *lainsoni* Silveira et al. 1987, *L.* (*V.*) *shawi* Lainson et al. 1989, *L.* (*V.*) *naiffi* Lainson and Shaw 1989, and *L.* (*V.*) *lindenbergi* Silveira et al. 2002 [15, 18]. Human infections with these leishmanial parasites give rise to an extensive array of clinical and immunological manifestations that range from localized cutaneous leishmaniasis (LCL) at the center of the spectrum to mucocutaneous leishmaniasis (MCL) at the hypersensitivity pole and anergic diffuse cutaneous leishmaniasis (ADCL) at the hyposensitivity one [37, 38]. *L*. (*V*.) *braziliensis* tends to provoke infections with accentuated hypersensitivity (MCL), while in contrast those with *L.* (*L.*) *amazonensis* tend toward hyposensitivity (ADCL) [39, 40]. A *Leishmania* species may be transmitted by different sand fly (Diptera: Psychodidae: Phlebotominae) species in different geographical regions and biomes. *L.* (*V.*) *braziliensis* is a good example of a species that is transmitted by different vectors in different ecological niches throughout the Americas [35, 42]. Most studies are one-off and beyond the immediate study areas, little is known about the range of other vectors that are involved. However, a general picture is emerging of a mosaic of enzootic and zoonotic cycles for a given *Leishmania* species in different ecological niches that range from primary forest to highly anthropogenic areas [6].

In Brazil, the National Forests (abbreviated in Portuguese as “FLONA” – FLOresta NAcional) are conserved forested areas composed of predominantly native species. The goal is to create and maintain forests that are used for both scientific investigations and different methods of sustainable exploitation of the native flora [7]. These conserved areas contain a rich fauna of insects and mammals that are compatible with the establishment and maintenance of *Leishmania* life cycles [29]. Brazil has 65 registered FLONAs and 32 are located in the northern region of the country. Of this total 13 FLONAs are located in Pará State and those such as the FLONAs of Altamira, Carajás, Itaituba, and Tapajós are among the largest in the country, each having an area of over 400,000 ha.

The following six dermotropic *Leishmania* species, *L.* (*V.*) *braziliensis*, *L*. (*V.*) *guyanensis*, *L.* (*L*.) *amazonensis*, *L.* (*V*.) *lainsoni*, *L.* (*V*.) *shawi shawi*, *L.* (*V.*) *shawi santarensis* Jennings, Souza, Ishikawa, Shaw, Lainson & Silveira 2014 and a putative hybrid parasite, *L.* (*V*.) *guyanensis/L.* (*V.*) *shawi shawi* were identified in patients who had contracted the disease in the western region of Pará State [16]. Part of the area is composed of the Tapajós FLONA and some years ago the sand fly fauna was studied in an urban area on the outskirts of Santarém city [8], but there were no data on vector incrimination. The aim of the present study was to identify the phlebotomine sand fly fauna of the Tapajós FLONA, located in the lower Amazon region in western Pará State, Brazil, and to incriminate putative vectors by identifying flagellate infections of female flies.

## Materials and methods

### Study area

**Figure 1.**
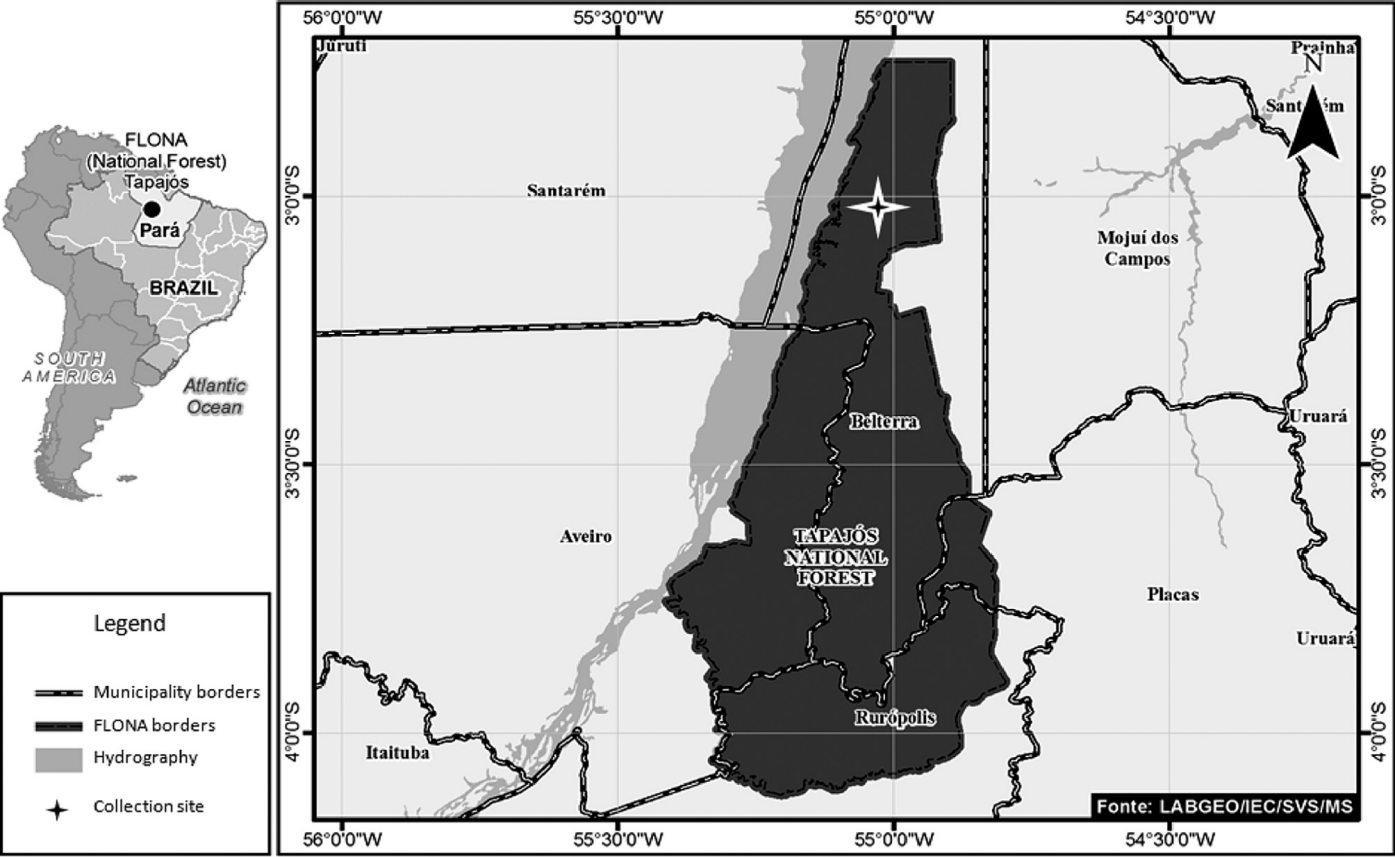
Study area. The location of Tapajós FLONA in the lower Amazon region, western Pará State, Brazil.

The Tapajós FLONA (20°45′ S 55°00′ W) (Fig. 1) occupies an area of 545,000 ha of predominantly dense rain forest. The climate is hot and humid with temperature variations between 21 °C and 31 °C, and over 2,000 mm of rain per year, relative humidity is above 80% and its altitude varies between 19 and 200 m above sea level. It straddles the lower Amazon municipalities of Belterra, Rurópolis, and Placas of western Pará State, Brazil. The actual study area was located within the FLONA area of Belterra municipality and situated 15 km from km 67 of the BR 163 highway [41].

### Sand fly collection and identification

Collections were made on six different occasions during a period of 10 days between 2002 (April, June, September, and November) and 2003 (May and October). Eight Centers for Disease Control (CDC) light traps were set between 18:00 and 06:00 hrs each night at 1.5 m ground level (6) and at 20 m in the canopy (2). A light-baited Shannon trap was also used between 18:00 and 20:00 as well as captures form the base of three trees from 07:00 to 09:00 with an aspirator. The sampling effort was calculated for each trapping method by multiplying the number of collections (55 for CDC, 52 for Shannon, and 17 for aspiration on tree bases) by exposure time (12 h for CDC and 2 h for Shannon and 2 h for aspiration on tree bases). Females were dissected under sterile conditions according to Ryan et al. [35] and the males were stored in 70% alcohol. All specimens were initially identified according to the pictorial key of Young & Duncan [51] and allotted to genera in accordance with the taxonomic criteria proposed by Galati [12] using the abbreviation suggested by Marcondes [26]. Material was also mounted in berlese liquid (GBI Laboratories) and deposited in the “Instituto Evandro Chagas” Phlebotominae sand fly collection. Species abundance was expressed for the four surveyed ecotopes (ground, canopy, Shannon, and tree bases collected) with the index of species abundance (ISA) and standard index of species abundance (SISA) as described elsewhere [34].

### Identification of *Leishmania* strains

The homogenate obtained from the digestive tract of naturally infected females was inoculated into two culture tubes containing Difco B^45^ media prepared according to Walton et al. [49]. The isolates were characterized with a panel of 23 monoclonal antibodies (B2, B5, B12, B11, B13, B18, B19, CO1, CO2, CO3, D13, L1, LA2, M2, N2, N3, V1, WA2, W1, W2, WH1, VIC.79.3, and T3) according to Shaw et al. [44]. Strains were also characterized phenotypically by isoenzyme electrophoresis for the 6-Phosphogluconate Dehydrogenase (_6_PGDH, EC 1.1.1. 44) enzymatic system, according to the method described by Miles et al. [27]. The McAb and/or electrophoretic profiles from the *Leishmania* isolates were compared with those of the World Health Organization (WHO) reference strains maintained in the cryobank of the Leishmaniasis Laboratory “Prof. Dr. Ralph Lainson”, as follows: *L.* (*L.*) *amazonensis* (IFLA/BR/67/PH8), *L.* (*V*.) *braziliensis* (MHOM/BR/75/M2903, *L*. (*V*.) *guyanensis* (MHOM/BR/75/M4147), *L.* (*V.*) *naiffi* (MDAS/BR/79/M5533), *L.* (*V*.) *lainsoni* (MHOM/BR/81/M6426), *L.* (*V.*) *s. shawi* (MCEB/BR/84/M8408), and *L.* (*V*.) *lindenbergi* (MHOM/BR/98/16714). A strain of the recently ranked subspecies, *L.* (*V.*) *shawi santarensis* (MHOM/BR/90/M13070), was taken into consideration as a novel *Leishmania* reference strain, but not included in the electrophoretic run as its _6_PGDH profile is the same as that of the *L.* (*V.*) *s. shawi* WHO reference strain.

## Results

### Sand fly fauna

A total of 9,704 individuals were collected of which 6,179 were females and 3,525 males, belonging to 68 species or subspecies, as shown in Table 1. Most individuals were collected in 660 h/trap hours in ground level CDCs (3,909), followed by 110 h Shannon trapping (3,399), 660 h/trap canopy CDCs (1,753), and 34 h of aspiration on tree bases (643).

**Table 1.** Species composition of the Phlebotominae sand fly fauna of Tapajós National forest (Tapajós FLONA), in the Lower Amazon region, western Pará State, Brazil (2002–2003).

*S*	Species	CDC ground		CDC canopy		Shannon		Tree bases		Total	%	Abundance		
		F	M	F	M	F	M	F	M			ISA	SISA	Rank
1	*Ps.complexus/wellcomei* *	59	17/23	58	2/3	1114**	52/62	–	–	1390	14.3	8.25	0.827	4th
2	*Ny. umbratilis* *	173	26	340	63	326	16	20	16	980	10	3.25	0.946	1st
3	*Th. ubiquitalis* *	170	510	27	28	23	21	7	2	788	8.1	7.75	0.839	3rd
4	*Ny. whitmani* * (6)	170 (3)	66	161 (1)	210	105 (2)	2	7	29	750	7.7	4.25	0.922	2nd
5	*Th. castanheirai*	55	565	6	40	12	8	–	–	686	7	17.75	0.601	15th
6	*Ps. davisi* * (2)	63	40	36	5	288 (2)**	62	1	–	495	5.1	8.75	0.815	5th
7	*Mi. rorotaensis*	59	21	34	–	9	15	45	255	438	4.5	10.75	0.767	7th
8	*Ny. shawi* (1)	94 (1)	5	99	1	87	3	–	1	290	2.9	9.5	0.797	6th
9	*Ps. paraensis* *	21	15	20	4	134	82	–	1	277	2.8	13	0.714	10th
10	*Ps. c. carrerai*	16	5	48	7	142	47	–	–	265	2.7	20.5	0.535	18th
11	*Ps. leonidasdeanei*	26	6	5	–	196**	11	–	–	244	2.5	23.5	0.464	25th
12	*Ny. anduzei* *	26	7	50	69	85	2	1	–	240	2.4	12.25	0.732	9th
13	*Ty. longispina*	4	216	3	3	–	–	1	–	227	2.3	21.25	0.517	20th
14	*Ev. saulensis*	186	6	12	–	4	–	–	–	208	2.1	23.75	0.244	38th
15	*Vi. furcata*	67	13	31	38	9	1	13	–	172	1.7	11.75	0.744	8th
16	*Ev. infraspinosa* (1)	59 (1)	91	12	–	5	2	–	–	169	1.7	23.25	0.47	24th
17	*Ps. corossoniensis*	29	–	7	–	120	–	–	–	156	1.6	23.75	0.458	26th
18	*Ev. campbelli*	2	137	2	1	–	1	3	–	146	1.5	19.5	0.559	16th
19	*Lu. gomezi* (1)	82	7	36	2	15 (1)	–	2	–	144	1.4	13.5	0.702	11th
20	*Pi. damascenoi*	32	–	13	–	7	1	8	67	128	1.3	14.25	0.684	12th
21	*Bi. flaviscutellata* *	61	13	23	–	24	3	4	–	128	1.3	16.25	0.636	14th
22	*Ps. h. hirsutus* (1)	9	7	34 (1)	2	57	17	–	–	126	1.2	15.75	0.648	13th
23	*Ty. dasypodogeton*	79	24	7	3	7	1	4	1	126	1.2	23.75	0.458	26th
24	*Ny. richardwardi*	23	22	10	–	51**	12	–	–	118	1.2	22.5	0.488	23th
25	*Ny. antunesi*	50	2	37	–	6	–	–	–	95	0.9	23.75	0.458	26th
26	*Br. avellari*	–	78	–	9	–	2	–	–	89	0.9	26.5	0.392	28th
27	*Mi. trinidadensis*	16	3	9	1	1	–	9	47	86	0.8	20	0.547	17th
28	*Ps. claustrei*	5	2	7	–	31	15	–	–	60	0.6	28.5	0.345	32th
29	*Br.* spp.	43	–	10	–	1	–	–	–	54	0.5	20.75	0.529	19th
30	*Pa. dendrophyla*	2	1	11	2	3	4	11	19	53	0.5	21.75	0.505	21th
31	*Pa. aragaoi*	17	25	3	6	–	–	–	–	51	0.5	31	0.285	35th
32	*Sc. sordellii* (2)	32 (2)	3	2	5	1	5	–	–	48	0.4	27.75	0.363	29th
33	*Pr. trispinosa*	–	24	–	4	–	14	–	–	42	0.4	28	0.357	30th
34	*Mg. migonei*	6	5	8	16	–	1	–	–	36	0.3	29.5	0.321	33th
35	*Ev. evandroi*	17	–	18	–	1	–	–	–	36	0.3	30.25	0.303	34th
36	*Vi. tuberculata*	18	1	7	3	5	–	–	1	35	0.3	22	0.5	22th
37	*Ps. bispinosus*	2	–	3	–	26	4	–	–	35	0.3	31.25	0.279	36th
38	*Pa. scaffi*	3	–	–	–	1	2	–	24	30	0.3	29.5	0.321	33th
39	*Pa. dreisbachi*	22	–	5	–	–		2	–	29	0.2	26.5	0.392	28th
40	*Pa. shannoni*	–	1	1	–	6	1	10	8	27	0.2	25.5	0.416	27th
41	*Pi. serrana*	3	13	1	–	1	–	–	2	20	0.2	25.5	0.416	27th
42	*Lu. carvalhoi*	13	–	4	2	–	–	–	1	20	0.2	28.25	0.351	31th
43	*Ps. geniculatus*	1	–	7	1	11	–	–	–	20	0.2	31.75	0.267	37th
44	*Mi. pilosa* (4)	14 (3)	–	2	–	–	–	3 (1)	–	19	0.1	28.5	0.345	32th
45	*Mi. oswaldoi*	11	–	1	–	–		3	–	15	0.1	28.5	0.345	32th
46	*Ev. sericea*	8	7	–	–	–	–	–	–	15	0.1	29.5	0.321	33th
47	*Pa. lutziana*	–	10	3	2	–	–	–	–	15	0.1	39.75	0.077	50th
48	*Ps. amazonensis*	–	–	–	2	7	2	–	1	12	0.1	37.75	0.125	43th
49	*Ty. trichopyga*	9	–	–	–	–	–	1	1	11	0.1	33.75	0.22	39th
50	*Pa. punctigeniculata*	–	–	–	–	–	1	3	5	9	–	31.75	0.267	37th
51	*Br. travassosi*	–	6	–	1	–	–	–	–	7	–	37.75	0.125	43th
52	*Mi. longipennis*	5	–	1	–	–	–	–	–	6	–	38	0.119	44th
53	*Pi. nevesi*	4	–	1	–	–	–	–	–	5	–	38.25	0.113	45th
54	*Pa. servulolimai*	–	3	–	–	–	2	–	–	5	–	39	0.095	47th
55	*Ev. bacula*	2	1	–	–	2	–	–	–	5	–	39	0.095	47th
56	*Ev. begonae*	2	–	–	2	–	–	–	–	4	–	35.25	0.184	40th
57	*Pr. triacantha*	3	–	–	–	–	–	1	–	4	–	38.5	0.107	46th
58	*Ps. ayrozai* *	1	2	–	–	–	–	–	–	3	–	42.25	0.017	52th
59	*Pa. abonnenci*	–	–	–	–	–	–	–	2	2	–	35.75	0.172	41th
50	*Ev. wilsoni*	–	2	–	–	–	–	–	–	2	–	42.5	0.011	53th
61	*Ps. s. squamiventris* *	–	–	–	–	1	–	–	–	1	–	36	0.166	42th
62	*Lu. spatotrichia*	1	–	–	–	–	–	–	–	1	–	39.25	0.089	48th
63	*Pa. inflata*	–	–	1	–	–	–	–	–	1	–	40	0.071	51th
64	*Pi. monticola*	–	–	–	–	–	–	1	–	1	–	40	0.071	51th
65	*Ev. monstruosa*	1	–	–	–	–	–	–	–	1	–	42.75	0.005	54th
66	*Ev. pinottii*	1	–	–	–	–	–	–	–	1	–	42.75	0.005	54th
67	*Ps. lainsoni*	–	–	–	–	1	–	–	–	1	–	42.75	0.005	54th
68	*Ps. s. maripaensis*	1	–	–	–	–	–	–	–	1	–	42.75	0.005	54th
	Total	1878	2031	1216	537	2925	474	160	483	9704				

*S*: Taxa; F: Females; M: Males; ISA: Index of Species Abundance; SISA: Standard Index of Species Abundance;

* Species already associated to American cutaneous leishmaniasis agents in the Amazon region;

** Some specimens found attempting to bit collectors; (*n*) Number of individuals found with natural infection by flagellates; n/n Expressed number for males of *Ps. complexus* and *Ps. wellcomei*, respectively.

Fifteen genera were identified and are as follows: *Psychodopygus* (*Ps*., 16 spp.), *Psathyromyia* (*Pa.*, 10 spp.), *Evandromyia* (*Ev.*, 10 spp.), *Nyssomyia* (*Ny.*, 6 spp.), *Micropygomyia* (*Mi.*, 5 spp.), *Pintomyia* (*Pi.*,4 spp.), *Trichopygomyia* (*Ty.*, 3 spp.), *Lutzomyia* (*Lu.*, 3 spp.), *Trichophoromyia* (*Th*.,2 spp.), *Viannamyia* (*Vi*., 2 spp.), *Pressatia* (*Pr.*, 2 spp.), *Brumptomyia* (*Br*., 2 spp.), *Bichromomyia* (*Bi.*, 1 spp.), *Migonemyia* (*Mi.*, 1 spp.), and *Sciopemyia* (*Sc.*,1 spp.).

The most frequent sand fly species found were: the closely related *Ps. complexus/Ps. wellcomei* (14.3%, SISA Rank 4th), followed by *Ny. umbratilis* (10.0%, SISA Rank 1st), *Th. ubiquitalis* (8.1%, SISA Rank 3rd) and *Ny. whitmani* (7.7% SISA Rank 2nd).

### Known vectors

Twelve species known as incriminated or suspected vectors of ACL agents in Amazonian Brazil were identified with their respective frequency, as follows: *Ps. complexus/Ps. wellcomei* (1,231 females, 71 *Ps. complexus* and 88 *Ps. wellcomei* males), *Ny. umbratilis* (859 females and 121 males), *Ny. whitmani* (443 females and 307 males), *Ps. davisi* (388 females and 107 males), *Th. ubiquitalis* (227 females and 561 males), *Ny. anduzei* (162 females and 78 males), *Ps. paraensis* (175 females and 102 males), *Bi. flaviscutellata* (112 females and 16 males), *Ps. ayrozai* (1 female and 2 males), and *Ps. squamiventris sensu latu* (2 females).

### Natural infections

Natural flagellate infections were found in 18 of 6,179 dissected females (infection rate: 0.29%) that belonged to the following eight species (Table 2): *Ny. whitmani* (6/486, 3 from CDC ground, 1 from CDC canopy, and 2 from Shannon), *Mi. pilosa* (4/19, 3 from CDC ground and 1 from CDC canopy), *Ps. davisi* (2/388, from Shannon), *Sc. sordellii* (2/35, from CDC ground), *Lu. gomezi* (1/135, from Shannon), *Ps. h. hirsutus* (1/100, from CDC canopy), *Ev. infraspinosa* (1/76, from CDC ground), and *Ny. shawi* (1/280, from CDC ground).

**Table 2.** Natural *Leishmania* (*Viannia*) spp. infections in phlebotomine sand flies isolated and characterized by monoclonal antibodies.

N	Sand fly species	Collection	Development	Result	MCAb reaction-profile	WHO code
1	*Ps. h. hirsutus*	CDC canopy	Peripylaric	*Leishmania* (*Viannia*) *naiffi*	B2, B12, L1, N2, N3	IHIR/BR/2002/M20906
2	*Ps. davisi*	Shannon	Peripylaric	*L.* (*V*.) *naiffi*	B2, B12, L1, N2, N3	IDAV/BR/2002/M20905
3	*Mi. pilosa*	CDC ground	Peripylaric	negative/contamined	–	–
4	*Ny. whitmani*	CDC ground	Peripylaric	*L.* (*V.*) *shawi shawi*	B2, B12, L1	IWHI/BR/2002/M21354
5	*Mi. pilosa*	CDC ground	Peripylaric	negative/contamined	–	–
6	*Ny. whitmani*	Shannon	Peripylaric	negative/contamined	–	–
7	*Ny. whitmani*	Shannon	Peripylaric	negative/contamined	–	–
8	*Sc. sordellii*	CDC ground	Peripylaric	non *Leishmania* Trypanosomatidae	–	ISOR/BR/2002/M21358
9	*Mi. pilosa*	CDC ground	Hipopylaric	negative/contamined	–	–
10	*Mi. pilosa*	Tree bases	Hipopylaric	negative/contamined	–	–
11	*Ny. shawi*	CDC ground	Peripylaric	negative/contamined	–	–
12	*Sc. sordellii*	CDC ground	Peripylaric	negative/contamined	–	
13	*Ps. davisi*	Shannon	Peripylaric	*L.* (*V.*) *naiffi*	B2, B12, L1, N2, N3	IDAV/BR/2003/M21903
14	*Ny. whitmani*	CDC ground	Peripylaric	negative/contamined	–	–
15	*Ev. infraspinosa*	CDC ground	Peripylaric	negative/contamined	–	–
16	*Ny. whitmani*	CDC ground	Peripylaric	*L.* (*V.*) *s. shawi*	B2, B12, L1	IWHI/BR/2003/M22342
17	*Ny. whitmani*	CDC canopy	Peripylaric	*L.* (*V.*) *s. shawi*	B2, B12, L1	IWHI/BR/2003/M22341
18	*Lu. gomezi*	Shannon	Peripylaric	*L.* (*V.*) *s. shawi*	B2, B12, L1	IGOM/BR/2003/M22340

McAb: Monoclonal Antibody; WHO: World Health Organization.

Flagellates from eight infections were successfully isolated in culture media (Difco B^45^) and eight were *Leishmania* spp. and one was a non-*Leishmania* Trypanosomatidae parasite. Four strains from *Ny. whitmani* (2 from CDC ground and 1 from CDC canopy) and *Lu. gomezi* (1 from Shannon) were characterized as *L.* (*V.*) *s. shawi* and 3 from *Ps. h. hirsutus* (1 from CDC canopy) and *Ps. davisi* (2 from Shannon) were identified as *L.* (*V*.) *naiffi*.

The monoclonal antibody (McAb) reaction profiles of both characterized *L.* (*V.*) spp. (*L. s. shawi* and *L. naiffi*) were identical to those of their WHO reference strains, presenting positive reactions to McAb B2, B12, and L1 (McAb reaction profile IV) for *L.* (*V.*) *s. shawi* and B2, B12, L1, N2, and N3 for *L.* (*V.*) *naiffi*, respectively.

**Figure 2.**
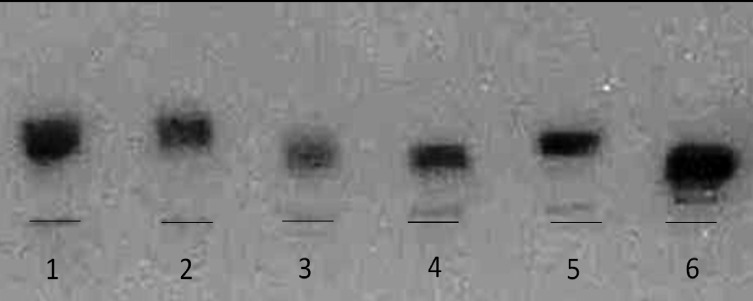
_6_PGHD isoenzyme electrophoresis analysis for identifying a *Leishmania* spp. strain isolated from *Ny. whitmani* collected in the Tapajós FLONA (Belterra municipality), lower Amazon region, western Pará State, Brazil. From left to right: 1 – *L.* (*V.*) *braziliensis* (MHOM/BR/75/M2903), 2 – *L.* (*V.*) *guyanensis* (MHOM/BR/75/M4147), 3 – IWHI/BRA/2002/M21354, 4 – *L.* (*V.*) *s. shawi* (MCEB/BR/84/M8408), 5 – *L.* (*V.*) *naiffi* (MDAS/BR/79/M5533), 6 – *L.* (*V.*) *lindenbergi* (MHOM/BR/98/16714).

The identity of *L.* (*V.*) *s. shawi* strains was confirmed by comparing their _6_PGDH profile with a strain isolated from *Ny. whitmani* whose isoenzymatic profile was identical to that of the *L.* (*V.*) *s. shawi* WHO reference strain (Fig. 2).

## Discussion

There are few studies that describe the phlebotomine sand fly fauna of Brazilian FLONA(s), especially in the Amazon region. These environments have an exuberant biodiversity that favors a variety of microclimatic conditions that are suitable for the development of immature sand flies and a rich vertebrate fauna that serves as a source of food for adult females. For example, 69 sand fly species have been recorded [46] in the “Serra dos Carajás” (southeast Pará State) in which the Tapirapé-Aquiri FLONA is located.

There is an inevitable bias in the species composition of catches according to the trapping method. One of the challenges is to assess which method best reflects the composition of the sand fly fauna in a given biome or ecological niche. For instance, the three dominant species in CDC ground traps were *Ny. umbratilis*, *Th. ubiquitalis*, and *Ny. whitmani* yet in the Shannon trap *Ps. wellcomei/complexus* females dominated. However, although the Shannon trap is a light trap, there is a human bait element of the person who is collecting the flies as they alight on the trap’s surface material. Of the total females captured in ground level CDCs, 3.1% of the females were *Ps. wellcomei/complexus* while in the Shannon trap these same females represented 38%. In the CDC ground level trap, 12% of the female flies belonged to the genus *Psychodopygus* and 28.5% to the genus *Nyssomyia* but in the Shannon trap 72.6% of the females were *Psychodopygus* and 22.4% were *Nyssomyia*. The question is: which trapping method gave the best representation of the leishmaniasis vectors in our study area? It is tempting to say that without a doubt it was the Shannon trap in which *Psychodopygus* species were overwhelmingly dominant, especially *Ps. complexus/Ps. wellcomei*. However, no infections were found in 1,231 females of this group and 4 of the 7 *Leishmania* infections were found in flies that did not belong to this genus. Also, none of the infections belonged to the *braziliensis* complex and in a recent study [16] only 9.4% of the patients from this general area were infected with *L.* (*V.*) *braziliensis.* Overall, we captured 12 phlebotomine species that have been implicated as vectors of ACL agents in the Brazilian Amazon region. Their importance in relation to the Tapajós FLONA area is discussed below.

The complex of two cryptic species belonging to the *chagasi* series, *Ps. complexus/Ps. wellcomei*, was the most frequent *taxa* in the collections. *Ps. wellcomei* is undoubtedly considered the major vector of *L.* (*V*.) *braziliensis* in the Carajás region of the Brazilian Amazon [20, 32]. However, *Ps. complexus*, a closely related sand fly morphologically distinguishable from the former only by the males was also associated with the transmission of this parasite in areas where *Ps. wellcomei* is absent [45]. Unfortunately, due to their sympatric occurrence in the Tapajós FLONA and the lack of technical conditions for characterization of these specimens at specific level, it was not possible to avoid ambiguous identification, and thus they have been regarded in this study as a single species complex. Curiously, although 1,231 females of *Ps. complexus/Ps. wellcomei* were dissected to search for natural infection by their associated parasite *L.* (*V.*) *braziliensis*, none was found with flagellates. This leads us to suggest that the *Ps. complexus/Ps. wellcomei* females are feeding on animals that are not reservoirs of *L.* (*V*.) *braziliensis* or that these parasite reservoirs are perhaps present in small numbers, resulting in only sporadic transmission to man. Identification of the males suggests that these two species were present in similar numbers. However, in the Carajás, seven ecotopes were sampled with CDCs and in none were the proportion of males of the two species similar [32].

The second most frequent vector found in the Tapajós FLONA was *Ny. umbratilis*, which is considered to be the principal vector of *L.* (*V.*) *guyanensis* ACL in the region to the north of the Amazon River [1, 2, 22, 33]. Fraiha et al. [11], collecting anthropophilic sand flies along the Transamazonian Highway, have found this species in localities such as Km 211 of the Santarém-Cuiabá Highway, in the nearby area of Tapajós FLONA. More recently, Feitosa & Castellón [8] registered *Ny. umbratilis* as the fourth most frequent sand fly species in the outskirts of Santarém municipality. The closely related species, *Ny. anduzei*, was also identified in the Tapajós FLONA, but in low numbers and is also considered as a vector of *L.* (*V.*) *guyanensis* ACL [21, 50].

Early data on ACL etiology in the lower Amazon region linked *Ny. umbratilis* to *L.* (*V*.) *guyanensis* and Jennings et al. [16] recorded the presence of two isolates of this same parasite from Santarém municipality in the “leishmanian bridging zone” south of the Amazon River. No natural infection was found in a considerable number of Ny. *umbratilis* females but on epidemiological grounds, it must be considered as a potentially important vector in the studied area.

*Th. ubiquitalis* was the third most frequent vector found. Again no infections were found but Jennings et al. [16] identified 6 ACL *L.* (*V.*) *lainsoni* infections in patients from the same region. So far *Th. ubiquitalis* is the only known vector of *L.* (*V.*) *lainsoni* in Brazil [25, 36] so its presence in large numbers is consistent with the number of ACL cases with this parasite.

*Nyssomyia whitmani* deserves special attention since it was the fourth commonest vector species and 1% (5/486) of the females had flagellates. Four *Leishmania* strains were successfully isolated and characterized as *L.* (*V.*) *s. shawi*, reinforcing the well-known role of *Ny. whitmani* in the transmission of this parasite in the lower Amazon region of western Pará as well as in eastern Amazonian areas [23, 29]. All *L.* (*V.*) *s. shawi* strains characterized in this study were from *Ny. whitmani* sand flies collected by CDC light traps at ground and canopy level. The monoclonal antibody profile of these strains was identical to that of the WHO reference strain isolated from the non-human primate *Sapajus apella* (syn. *Cebus apella*) Linnaeus 1758. No natural infections of *L.* (*V.*) *s. santarensis* were found in *Ny. whitmani* or any other sand fly species so its vector(s) remains unknown.

*Leishmania* (*V.*) *guyanensis* has been recorded in *Ny. whitmani* from captures in localities of the northern bank of the Amazon River, such as Monte Dourado (Pará State) [22, 35] and Serra do Navio (Amapá State) (Souza, unpublished observation), but its role in the epidemiology of Guianan ACL needs to be evaluated in greater detail and will be discussed in our further publication. However, the fact that it has been found naturally infected with two different parasites of the *guyanensis* complex adds weight to the hypothesis that the putative hybrid parasite, *L.* (*V.*) *guyanensis/L.* (*V.*) *s. shawi*, is the result of genetic exchange between these two closely related *Leishmania* species that share the same sand fly vector, *Ny. whitmani* [16].

Besides being found in *Ny. whitmani*, *L.* (*V.*) *s. shawi* was also isolated from one specimen of *Lu. gomezi* from the Shannon trap. This species is implicated as a vector of *L.* (*V.*) *panamensis* in Panama [47] and other Latin American countries [9, 48]. It is conceivable that *Lu. gomezi* participates in the transmission of ACL in the Brazilian Amazon region as well as indicating a permissiveness of *L.* (*V.*) *s. shawi* in relation to its vectors. The association of this fly with arboreal animals, that are the reservoirs of this parasite, is also supported by the identification [10] of *Endotrypanum* spp., in a specimen of this fly captured in Rondônia [13].

Our finding of *L*. (*V.*) *naiffi* in *Ps. h. hirsutus* (1) and *Ps. davisi* (2), neither of which is considered as potential vectors of this viannian [29, 31], raises a number of intriguing possibilities. Its recognized vectors are from the *Psychodopygus panamensis* series, *Ps. paraensis*, *Ps. ayrozai* [3, 19] as well as suspected vectors within the *chagasi* series, such as *Ps. squamiventris s.l*. [14]. All these species were present in small numbers in our collections, suggesting that the enzootic cycle is present in the primary forest where it is being transmitted by various *Psychodopygus* species. However, Jennings et al. [16] failed to find *L.* (*V.*) *naiffi* in patients from the general area. Although *Ps. h. hirsutus* was uncommon in our catches, *Ps. davisi* was the third most frequent species in our Shannon trap catches. This suggests that transmission to man may be more frequent than is indicated and that the low-frequency figures [4, 16, 24, 28] could be partly explained by the benign nature of *L.* (*V.*) *naiffi* ACL.

In the 1980s trypanosomatid infections were found in 3 *Ps. h. hirsutus* captured in the Pará municipality of Tucuruí [35], and 1 from the Minas Gerais municipality of Além Paraíba [30]. At the time no specific monoclonal antibodies for *L.* (*V.*) *naiffi* were available and the parasites were identified as belonging to the *braziliensis* complex. However, a recent revision of these results shows that the B2 and B12 profiles of the four strains are identical to those of *L.* (*V.*) *naiffi* (Shaw unpublished observation). Five years later flagellates were found in two specimens of *Ps. h. hirsutus* from the Rondônia municipalities of Cacaulândia and Campo Novo [13]. Their serodemic profile, using the same panel of monoclonal antibodies as the present study, indicated that the parasites were *L.* (*V.*) *naiffi*. All these results suggest that *Ps. h hirsutus* is an important vector of *L.* (*V.*) *naiffi* throughout the forested regions of Brazil.

The fact that *Ps. h hirsutus* was collected in canopy CDC traps, including an infected one, might be interpreted as circumstantial evidence of an *L.* (*V.*) *naiffi* arboreal enzootic cycle involving other reservoirs. But *L.* (*V.*) *naiffi*’s accepted reservoir is the nine-banded armadillo, *Dasypus novemcinctus* Linnaeus 1758, which is an obligatory terrestrial mammal. On the other hand, it could just be a natural vertical migration of flies that became infected on the ground. An example of this is the vertical migration of *Ny. umbratilis* that becomes infected from the arboreal two-toed sloth but transmission to man occurs at ground level when the fly descends from the canopy during the day [33]. Using the k-DNA PCR technique, Cassia-Pires et al. [5] found evidence of *L.* (*V.*) *naiffi* in the caviomorph rodent *Thrichomys pachyurus* Wagner, 1845 (syn. *T. fosteri*) that lives in open areas of the caatinga and cerrado. Armadillos also live in this habitat so flies that fed on armadillos could have infected these rodents, or there is a separate cycle in these rats.

*Psychodopygus davisi* has already been incriminated as a potential vector of zoonotic *L.* (*V.*) *braziliensis* ACL in Rondônia State [14] and more recently in the Tapirapé-Aquiri FLONA, Pará State [46]. However, the present finding of two infections of *Ps. davisi*, one of which proved to be *L.* (*V.*) *naiffi* (Shannon collections), is in accordance with Gil et al. [13] who also found *Ps. davisi* to be naturally infected by *L.* (*V.*) *naiffi* in Rondônia State. These results strongly support the hypothesis that *Ps. davisi* is involved in the transmission of *L.* (*V.*) *naiffi* in both the eastern and western regions of the Brazilian Amazon.

Seven criteria based on epidemiological, entomological, parasitological, and mathematical data [17, 31] have been suggested for defining vector status. In our opinion the finding of diverse natural flagellate infections, on more than one occasion in *Ps. davisi* that were identical to the *Dasypus novemcintus* reference strain, and this fly’s epidemiological associations, achieves four of seven criteria. We therefore conclude that *Ps. davisi* should be regarded as a new suspected vector of the *L.* (*V.*) *naiffi* enzootic life cycle in the lower Amazon region.

The SISA ranking approximated a ranking based on the total number of individuals collected with a minor exception as *Ps. wellcomei/complexus* had a SISA ranking at 4th but it was 1st based on total numbers, probably biased due to the absence of this species in one out of four studied ecotopes (tree bases). However, all *Leishmania* infections, except one in *Ps. h. hirsutus*, occurred in flies ranked 10th or above. This suggests that the SISA ranking based on the overall number of flies captured in all traps gives a more accurate indication of the vectorial importance of each species than a simple ranking based on total numbers. In this respect it is interesting to note that of 43 strains isolated from patients who contracted the disease in the region of the study area, 11 were *L.* (*V*.) *braziliensis*, 6 were *L*. (*V.*) *lainsoni*, 2 were *L.* (*L*.) *amazonensis*, and 35 were species belonging to the *guyanensis* complex [16]. The latter are transmitted by two species of flies, *Ny. umbratilis* and *Ny. whitmani*, whose SISA ranking in our study is, respectively, 1 and 2. *L.* (*V.*) *naiffi*’s vector SISA ranking was 3rd but no human cases were recorded. However, the low pathogenicity of this parasite is thought to relate to cases being underreported. The species ranked 5th and 13th, *Ps. davisi* and *Ps. h. hirsutus*, are considered as vectors of *L.* (*V.*) *lainsoni* that was the 3rd most common parasite found in man after *L*. (*V*.) *braziliensis*, but there was no statistical difference between the numbers of cases of the two parasites.

Flagellate infections in *Ev. infraspinosa* and *Sc. sordellii* (syn. *Lu. nordestina*) were not isolated but past experience suggests that they have no importance in the epidemiology of leishmaniasis. These infections were trypanosomes and have previously been reported in these two sand fly species as far apart as Pará State and Rondônia [13, 35, 43]. The rondonian *Ev. infraspinosa* and *Sc. sordellii* infections proved to be the insect stage of a new clade of trypanosomes found in terrestrial anurans [10].

Our study has confirmed the presence of rich and varied anthropophilic sand fly fauna in the Tapajós FLONA that includes five sand fly species that are associated with *Leishmania* transmission. The finding of infections adds weight to the importance of *Ps. h. hirsutus* and *Ps. davisi* as vectors of *L.* (*V*.) *naiffi*, whereas the transmission of *L.* (*V.*) *s. shawi* is strongly associated with *Ny. whitmani*. The absence of *Leishmania* infections in *Ps. complexus/Ps. wellcomei*, *Ny. umbratilis*, and *Th. ubiquitalis* in no way diminishes their potential participation in the transmission of ACL in the lower Amazon region in western Pará State, especially as *L.* (*V*.) *braziliensis*, *L.* (*V.*) *guyanensis*, and *L.* (*V.*) *lainsoni* have been recorded in humans in the region [16]. Our results indicate the presence of complex and poorly understood ACL epidemiologies that are associated with very diverse sand fly fauna. Under these conditions humans can potentially become infected with more than one *Leishmania* species that may or may not result in different diseases.
